# From Planning to Practice: Impact of Achieved Proximal Sealing Zone in Endovascular Aneurysm Repair (EVAR)

**DOI:** 10.3390/jcm14041309

**Published:** 2025-02-16

**Authors:** Giulio Accarino, Angelo Silverio, Michele Bellino, Sergio Furgiuele, Mario Fimiani, Mattia Sica, Francesco De Vuono, Giovanni Fornino, Davide Turchino, Giancarlo Accarino, Raffaele Serra, Gennaro Galasso, Carmine Vecchione, Umberto Marcello Bracale

**Affiliations:** 1 Vascular Surgery Unit, Struttura Ospedaliera ad Alta Specialità Mediterranea, 80122 Naples, Italy; prof.furgiuele@gmail.com; 2 Department of Public Health, University of Naples “Federico II”, 80138 Naples, Italy; dott.turchino@gmail.com (D.T.); umbertomarcello.bracale@unina.it (U.M.B.); 3 Department of Medicine, Surgery and Dentistry, University of Salerno, 84081 Salerno, Italy; asilverio@unisa.it (A.S.); mbellino@unisa.it (M.B.); mariofimiani.mf@gmail.com (M.F.); msica@unisa.it (M.S.); francescodevuono98@gmail.com (F.D.V.); giancarloaccarin@me.com (G.A.); ggalasso@unisa.it (G.G.); cvecchione@unisa.it (C.V.); 4 Vascular and Endovascular Surgery Unit, Ospedale San Giovanni di Dio e Ruggi D’Aragona, 84131 Salerno, Italy; giovanni.fornino@sangiovannieruggi.it; 5 Department of Medical and Surgical Sciences, University “Magna Graecia” of Catanzaro, 88100 Catanzaro, Italy; 6 Interuniversity Center of Phlebolymphology (CIFL), International Research and Educational Program in Clinical and Experimental Biotechnology, University Magna Graecia of Catanzaro, 88100 Catanzaro, Italy; 7 Vascular Pathophysiology Unit, IRCCS Neuromed, 86077 Pozzilli, Italy

**Keywords:** EVAR, sealing zone, planning, guidelines

## Abstract

**Background:** Endovascular aneurysm repair (EVAR) is the preferred treatment for abdominal aortic aneurysms (AAAs). This study evaluated the differences between the anticipated and actual achieved proximal sealing zones for standard EVAR endografts and their potential implications in a real-world AAA population. **Methods:** Data from 275 consecutive EVAR patients treated with the Endurant endograft (Medtronic, Minneapolis, MN, USA) between 2009 and 2022 were retrospectively analyzed. The proximal sealing zone was calculated preoperatively (target anticipated sealing zone, TASZ) and postoperatively (real achieved sealing zone, RASZ) from computed tomography angiography (CTA) images. These metrics were evaluated by assuming that they had a truncated cone shape, calculating the cone’s lateral surface by measuring the proximal and distal centerline areas and the distance between the planes. The primary outcome was the occurrence of type 1A endoleak at the longest available follow-up. **Results:** RASZ was significantly smaller and shorter than TASZ (*p* = 0.001), with an average area reduction of 24.5 mm^2^ and a median length reduction of 3 mm. Area and cranial length loss were present even when correcting for graft positioning imperfections. In the Cox proportional hazard regression model, TASZ and RASZ lengths were both independently associated with a lower risk of type 1A endoleak (HR: 0.88, 95% CI 0.80–0.96 and HR: 0.92, 95% CI 0.86–0.99, respectively). A Kaplan–Meier analysis confirmed that patients with RASZ > 5.5 mm had a survival free from endoleak higher than patients with RASZ ≤ 5.5 mm. **Conclusions**: In this real-world AAA population, the achieved proximal sealing zone was significantly shorter and smaller than planned, regardless of optimal endograft placement. The early calculation of RASZ, i.e., the PSZ achieved via CTA, is critical for risk stratification and follow-up.

## 1. Introduction

Since its introduction three decades ago, endovascular aneurysm repair (EVAR) has become the treatment procedure of choice for most abdominal aortic aneurysms (AAAs) [1]. Its minimally invasive nature has enabled a significant reduction in hospital stay and recovery time over open surgery [2] and has profoundly broadened the number of patients that can be treated for AAAs despite pre-existing comorbidities [3]. The increased number of AAA surgeries has led to the spread of the EVAR procedure and an ever-increasing interest in the scientific community in perfecting the technique. While planning to treat an AAA, there is a consensus that both proximal and distal sealing zones are the most critical regions to evaluate in order to ensure better long-term outcomes [3,4]. The proximal sealing zone plays a critical role in the long-term durability of EVAR, as its integrity is closely linked to the risk of endoleaks and other adverse outcomes. Previous studies have emphasized the importance of achieving an adequate sealing zone to reduce the likelihood of complications such as type 1A endoleaks and sac expansion [4,5]. The proximal aortic sealing zone, commonly called the “neck”, is currently defined as the segment starting distal to the lowermost renal artery and ending at the distance where it shows an expansion of 10% of the initial diameter [6] and is measured in millimeters of length. In addition to length, other parameters should be evaluated, such as the degree of taper, width, angulation, and the presence of bulging. Current guidelines defer the feasibility of the intervention and device selection to individual manufacturers’ instructions for use (IFUs) and physician assessment [7]. Neck features should fall in the IFUs of the selected device, A 15/20% oversizing is usually applied to ensure enough radial force to seal the endograft to the aortic wall. This kind of evaluation tends to simplify the choice of device greatly and has proven successful to date; however, recent studies have already distinguished between a theoretical target anticipated sealing zone (TASZ) and a real achieved sealing zone (RASZ) [8]. RASZ can be influenced by anatomical and procedure-related factors such as suboptimal graft placement and endograft oversizing. 

This study’s primary aim was to introduce a reproducible method for quantifying the proximal sealing zone (TASZ and RASZ) pre- and postoperatively. We also aimed to evaluate the discrepancy between the theoretical sealing zone (TASZ) and the actual sealing zone (RASZ), even under optimal graft placement conditions. As an exploratory analysis, we investigated the association between sealing zone characteristics and the occurrence of type 1A endoleaks.

## 2. Materials and Methods

This was a monocentric retrospective observational study based on a registry of all consecutive patients subjected to EVAR with the Endurant endograft (Medtronic Vascular, Santa Rosa, CA, USA) between 2009 and 2022 at our institution. All data were irreversibly anonymized. All principles in the Declaration of Helsinki were adhered to and the Italian laws on privacy (Art. 20–21, DL 196/2003) as published in the *Official Journal*, volume 190, 14 August 2004, were respected. This study was approved by the local ethical committee “Campania 2” (n. 2024/21860). All patients gave written consent for the anonymous collection of clinical data using the standard consent form provided by our institution. The inclusion criteria were the presence of an AAA in need of treatment according to Italian guidelines, the adherence of the patient’s anatomy to the IFUs of the selected device, and the availability of pre- and postoperative computed tomography angiography (CTA) performed within 60 days of the procedure. Before the procedure, CTA was performed on our patients to assess the aorto-iliac anatomy and plan the procedure.

Procedural planning was carried out with dedicated software, and the feasibility of EVAR was discussed with the device specialist. Patients’ demographics, comorbidities, and anatomic characteristics were systematically collected. All procedures were performed in the same dedicated operating theater equipped with a GE OEC 9900 Elite C-arm (GE Healthcare, Little Chalfont, UK) in spinal anesthesia with surgical exposure of both common femoral arteries. All EVARs were performed using the Endurant endograft. Device descriptions and implantation techniques followed the standard of care previously described [9]. Procedural success was defined as seemingly successful endograft deployment in the planned position and the absence of type 1 o or 3 endoleaks. After the procedure, subjects were followed up on and underwent a CT angiography evaluation after 30 days. All CTAs were evaluated by the same physician during patient follow-up. Our patients were divided into two groups according to the development of an endoleak later in the follow-up: type 1A endoleaks and other endoleaks were identified by looking at postoperative CT scans, if available, or relying on follow-up reports available at our institutions. Group 1 was named “no endoleak”, and group 2 was named “endoleak”. In addition to introducing the proposed method, this study aimed to investigate the association between sealing zone parameters and late endoleaks.

### 2.1. Proximal Sealing Zone Assessment

Our technique was based on measuring the sealing areas of each patient from the CTAs in our database; all patients whose CTAs did not meet the requirements for resolution and proximity between the slices (512 × 512 pixels and 1 mm thickness) were excluded. All measurements were performed by the same physician, who did not operate on the patients, with high specialization, using version 10.3 3 mensio (Pie Medical Imaging, The Netherlands). The evaluator followed a standardized and predefined protocol for assessing CTAs based on objective and well-defined criteria. Furthermore, the evaluator was unaware of any study-specific assumptions during the evaluation process

To ensure the reliability of the assessments, we also subjected a random sample of the CTAs to an independent review by a second expert, obtaining interobserver variability with a Cohen kappa coefficient of 0.78, indicating good agreement (*p* < 0.001), confirming the accuracy and consistency of the measurements.

### 2.2. Measurement Protocol

Because the aorta often tends to deform so that it is not round, a diameter measurement may not be accurate, just as choosing multiple diameters to draw a complex figure may not be reproducible. Our study used the area drawn manually as a closed polygon utilizing the program’s built-in function.

### 2.3. Preoperative Assessment (TASZ)

**Figure 1:**
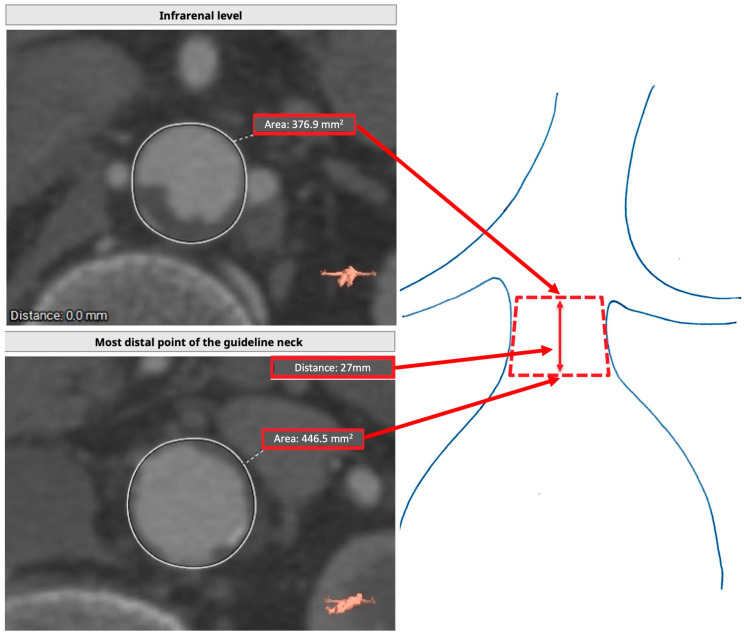
Target anticipated sealing zone measuring technique. The area of the infrarenal level is considered as the top area of a truncated cone. In contrast, the most distal point of the guideline neck is considered the lower area. The distance between the two planes is considered the height of the figure.

After obtaining the aorta’s center lumen line (CLL), the area immediately caudal to the lowest renal artery level was obtained using the closed polygon function. The diameter was also taken. The traditional way of evaluating the proximal aortic neck was carried out, searching for the point at which the diameter reached a 10% increase over the diameter of the infrarenal level. At this point, the area was obtained, and the CLL distance between the two planes was marked. Calcium and thrombus were treated as advised by current guidelines and endograft IFUs. In Figure 1, the TASZ is the lateral surface of the truncated cone between the two planes, which are the lower and the upper base, and the CLL distance is the height of the figure. Infrarenal neck angulation was primarily measured in the sagittal plane, as per standard EVAR planning practice. In cases where the coronal angulation exceeded the sagittal angulation, the coronal value was considered to better represent the anatomical complexity of the proximal landing zone.

### 2.4. Postoperative Assessment (RASZ)

**Figure 2:**
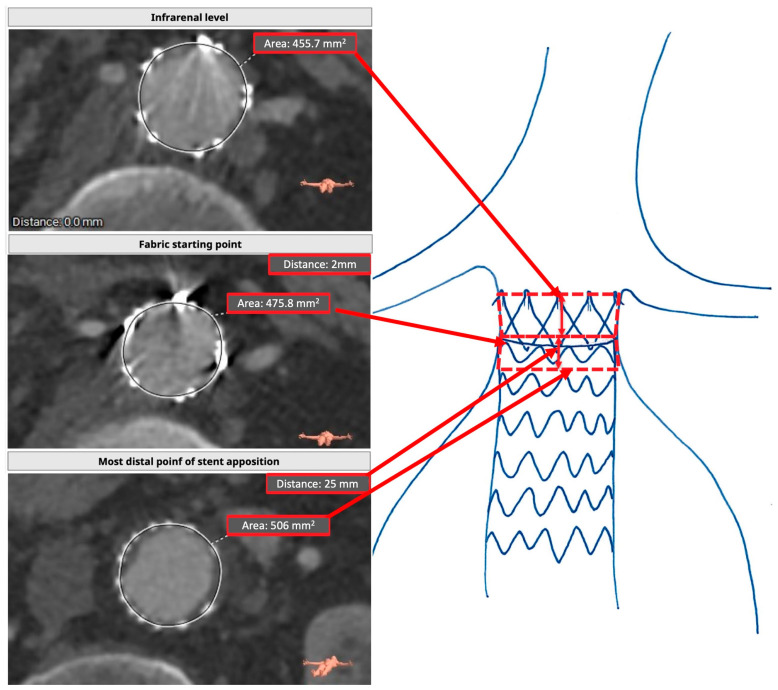
Real achieved sealing zone assessment. The area of the infrarenal level is considered as the top area of a truncated cone while the fabric starting point (if placed lower) is considered the lower area. The fabric starting point is the upper area of the lower truncated cone, where the lower is the last point of complete stent apposition. The distance between the three planes is considered the height of the figures.

After obtaining the CLL of the aorta, the area immediately caudal to the lowest renal artery level was obtained again using the closed polygon function. The area of the fabric starting point of the endograft, which was evident by the presence of gold markers, was also obtained. The last area that was taken was at the last point of circumferential stent apposition. The CLL distance between each of these three areas was also obtained. The area was drawn using the middle point of the stents as a reference, as we observed a slight penetration of the stents inside the aortic wall. In the postoperative assessment, we distinguished two truncated cones: one starting at the lowest renal artery level and ending at the fabric starting point, and one starting at the graft starting point and ending at the last point of complete stent apposition. The lower truncated cone was the real achieved sealing zone, while the upper one was the “lost” sealing area. If a graft was perfectly positioned, the fabric starting point corresponded to the area immediately caudal to the lowest renal artery, and the “upper truncated cone” was absent. When correcting for graft misplacement, the proximal sealing zone was measured starting at the lowermost renal artery, including the upper truncated cone. The endograft mechanically influenced the “lost” sealing area of the aorta for the presence of the free flow, which exerted a constant radial force on the aortic wall, so it was also considered. However, it was excluded from the sealing zone evaluation (Figure 2). We referred to the “lost” sealing area by measuring its length in millimeters, as it was easier to perform and more reproducible.

### 2.5. Statistical Analysis

The normal distribution of continuous parameters was tested with the Kolmogorov–Smirnov and the Shapiro–Wilk tests. Normally distributed variables were expressed as mean ± standard deviation and compared using the Student *t*-test; variables with a skewed distribution were reported as a median and interquartile range and were compared with the Mann–Whitney U test. Categorical variables were reported as numbers and percentages and compared using the χ^2^ test, or the Fisher exact test, when the number of observations in any row was lower than 5. The association between TASZ and RASZ was evaluated using paired-sample t-tests or a Wilcoxon test when appropriate. Univariable COX proportional hazard regression models were applied to assess the association between anatomical parameters and the risk of type 1A endoleak. All variables significantly associated with the risk of type 1A endoleak and not showing multicollinearity were entered into two multivariable Cox regression models to test the association between TASZ and RASZ lengths, respectively, with the risk of type 1A endoleak. The results were reported as hazard ratios (HRs) with their 95% confidence intervals (CIs); the hazard ratios (HRs) represented the risk of type 1A endoleak per each unit (mm or mm^2^). Receiver operating characteristic (ROC) curve analysis was performed to identify the best cutoff values of RASZ length for predicting type 1A endoleaks. Survival free from type 1A endoleaks was estimated using the Kaplan–Meier method, and the log-rank test was used to compare groups.

The association between oversizing and the difference between RASZ and TASZ length were evaluated using linear regression analysis. For all tests, a *p*-value < 0.05 was considered statistically significant. The analysis was performed using SPSS version 28.0 (SPSS Inc., Chicago, IL, USA) and JASP software 0.18.3 (JASP Team, Amsterdam, The Netherlands).

## 3. Results

The study population included 275 patients, with 91.9% males (mean age 72.2 ± 8 years), 213 in group 1 (no endoleak) and 62 in group 2 (endoleak). Table 1 summarizes the patient demographics and comorbidities.

Concerning the anatomical features listed in Table 2, AAA, aortic bifurcation, and access iliac artery diameter were larger in the endoleak group but infrarenal neck angulation did not differ between groups.

The procedural time was 3.0 ± 1.6 h. Proximal area-based oversizing (ratio of the neck area to the aortic graft area) was 1.5 ± 0.12- embolizing spirals were used in 22% of patients and a mean of three spirals was used.

Ten patients were treated in an emergency setting because of a symptomatic aneurysm, either ruptured (4/10) or with an impending rupture (6/10). No patient required open surgical conversion, and no intra-operative or postoperative complications secondary to endograft deployment occurred. Procedural success was achieved in 100% of cases. The median hospitalization time was 4 (3–5) days (Table 3). During a median follow-up of 64 (31–106) months, a total of 62 endoleaks were reported: 24 type 1A, 11 type 1B, and 27 type 2 after 18 (2–70) months. Outcomes for the whole cohort are presented in Table 4.

TASZ was significantly higher than RASZ (106 vs. 78 mm^2^, *p* < 0.001). The paired pre- and postoperatively assessed infrarenal level area (*p* = 0.530) and the projected most distal point of the guideline neck area/most distal point of circumferential stent apposition area (*p* = 0.374) were similar. The TASZ length corrected for graft misplacement was significantly higher than the RASZ length (15 vs. 11 mm, *p* = 0.001). RASZ and TASZ paired statistics are summarized in Table 5.

When comparing areas delimitating the PSZ in Table 6 (expected and achieved infrarenal level area and most distal point of neck/stent apposition area), we found no statistically significant differences. Patients from group 2 that developed a type 1A endoleak had an overall higher rate of low graft misplacement (*p* = 0.026), where the graft positioning was suboptimal and below the infrarenal level. 

**Figure 3:**
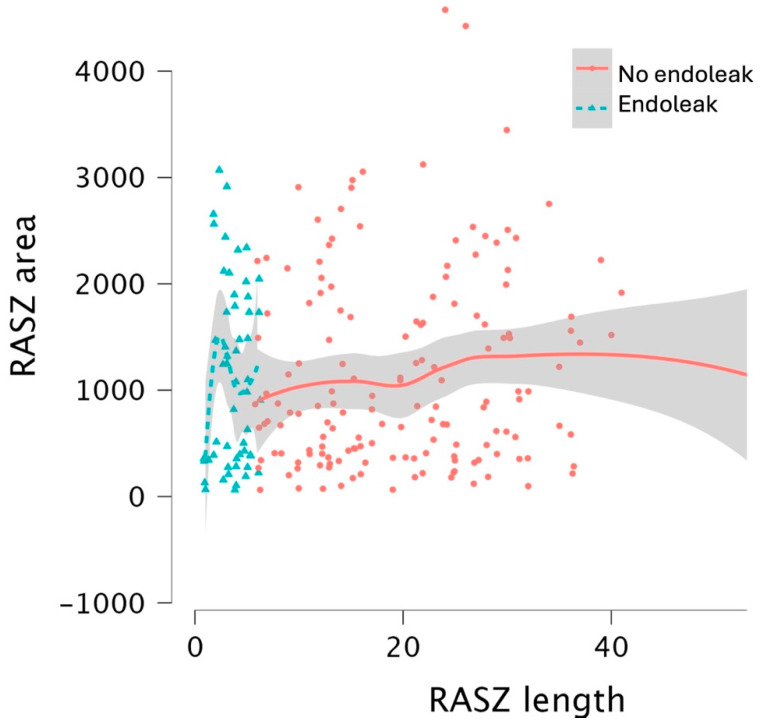
Scatterplot showing the distribution of RASZ area and length between the two groups. The endoleak group shows a lower overall RASZ length.

The average loss of sealing area between TASZ and RASZ was 24.5 mm^2^, which is consistent between the two groups. A scatterplot showing the distribution of RASZ area and length in the two groups is provided in Figure 3.

**Figure 4:**
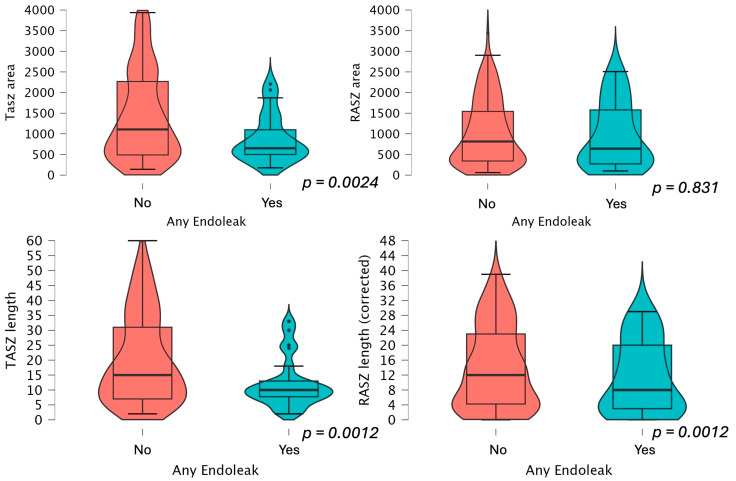
TASZ and RASZ area and length comparison between patients presenting an endoleak later in the follow-up.

**Figure 5:**
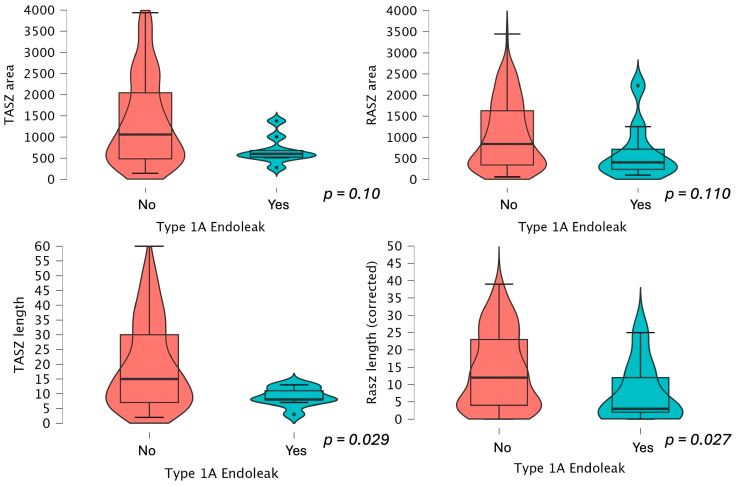
TASZ and RASZ area and length comparison between patients presenting a type 1A endoleak later in the follow-up.

RASZ and TASZ area and length measurements are presented in Figure 4 and Figure 5. The RASZ was shorter also in patients who developed a type 1A, 1B, and 2 endoleak later in the follow-up. The median RASZ length loss corrected for graft misplacement was 3 mm (−12.3), 7 mm (−14.1) if not corrected. The oversizing difference was not statistically significant between the two study groups, also when selectively evaluating type 1A endoleaks (*p* = 0.086). Oversizing did not influence the area of the most distal point of circumferential stent apposition and did not appear to be statistically correlated with a reduced sealing length loss (*p* = 0.671).

The univariable COX proportional hazard regression models found that the lost RASZ length and aortic bifurcation diameter differences were nonsignificant for type 1A endoleak risk over time (Table 7).

In the multivariable Cox regression analyses (Table 8), both TASZ length (HR: 0.88, 95% CI: 0.80–0.96, *p* = 0.003) and RASZ length (HR: 0.92, 95% CI: 0.86–0.99, *p* = 0.020) were independently associated with a lower risk of endoleaks.

ROC analysis identified 13.5 mm as the best cutoff length for TASZ for predicting type 1A endoleaks (AUC = 0.703 *p* < 0.001). Meanwhile, ROC analysis identified an RASZ length of 5.5 mm as the best cutoff value for predicting type 1A endoleaks (AUC = 0.727). Finally, Cox regression analysis confirmed that RASZ > 5.5 mm was associated with a lower risk of type 1A endoleaks (HR = 0.24, 95% CI 0.10–0.56, *p =* 0.001) and all endoleaks (HR = 0.67, CI = 0.61–0.74, *p* < 0.001).

**Figure 6:**
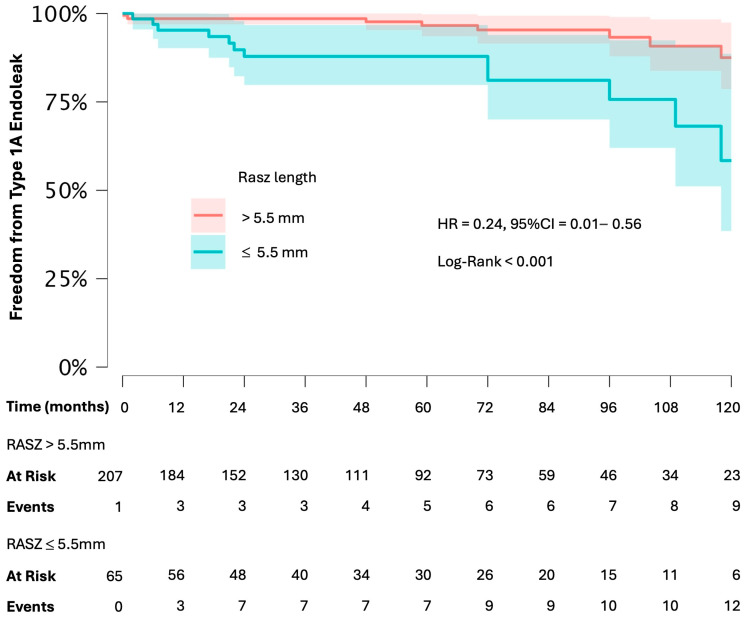
Freedom from type 1A endoleak in patients with RASZ length ≤ 5.5mm and >5.5mm, according to the Kaplan–Meier survival analysis.

In the Kaplan–Meier analysis, survival free from type 1A endoleaks was significantly lower in patients with RASZ ≤ 5.5 mm compared to those with RASZ < 5.5 mm (Figure 6).

## 4. Discussion

While EVAR has been increasingly established as the first-line therapy for most patients with AAA [10,11], there are still concerns about its long-term durability. The patient’s anatomy still plays an important role in the clinical outcomes [12,13]. To the best of our knowledge, this is the largest real-world study evaluating TASZ and RASZ measures. We included 275 consecutive patients treated with the same endograft and monitored them over a very long follow-up time. We excluded patients treated using other devices to avoid the confounding factor of different devices having different performance profiles.

We aimed to compare the components of the sealing zone before and after surgery and assess the clinical significance of each measure using open-source software and methods commonly used in EVAR planning.

The main results of this study can be summarized as follows:

- TASZ and RASZ were markedly different;
- The primary factor contributing to the difference between TASZ and RASZ was the length of the sealing zone. This variation continued even after considering graft misplacement;
- Oversizing did not affect RASZ length;
- Short RASZ and TASZ values were effective predictors of type 1A endoleaks during follow-up.

The paramount importance of the PSZ in preventing adverse events and the need for reintervention following an EVAR procedure is well established, as the insufficient sealing zone greatly increases endoleak risk, and could ultimately lead to post-EVAR aneurysm rupture [4]. Several hostility criteria have been proposed, and tested and are now considered by each operator planning an EVAR case [14,15]. RASZ measuring has been proposed as an important measure to keep during the first follow-up CTA to stratify each patient’s type 1A endoleak risk. Still, results showing its usefulness were mixed and the prognostic role of this parameter has not been clarified yet [4,16]. The proximal sealing zone should be large enough to allow the endograft to give enough radial force to seal the aorta, but the exact surface needed is only expressed as a length in the IFUs. Recent studies, such as the one by Sandström et al. (2024), further highlight that sealing zone failure significantly impacts the long-term durability of EVAR, reinforcing the importance of precise planning and evaluation of sealing zones [17]. Our findings are specific to the Endurant stent graft, which uses nitinol-based radial force for fixation. Devices with different sealing technologies, such as polymer-based or active seal mechanisms, may exhibit distinct sealing dynamics. These differences underscore the need for further research to evaluate how proximal sealing zone characteristics and their clinical implications vary across stent graft models. Our results should not be generalized beyond devices with fixation technologies similar to those of Endurant. In our cohort of patients, in addition to the PSZ there are several statistically significant differences in the group with endoleaks regarding other anatomical factors, such as the size of the aneurysm (67 vs. 57 mm *p* > 0.001), the external iliac artery, and aortic bifurcation. Our multivariate analysis highlighted the key role of covered length and postoperative distal sealing distance in predicting the risk of type 1A endoleaks after EVAR. The finding that a greater RASZ length was associated with a reduced risk aligns with the existing literature on the importance of the sealing zone in preventing complications. Aneurysm size has been proposed before as a risk factor for adverse events [18,19], but our multivariable analysis found it to be nonsignificant. In 2018, a study speculated that changes in aortic anatomy before an endoleak are happening over time and a near postoperative RASZ measurement may not be sufficient to foresee a type 1A endoleak later in the follow-up [20]. A recent study found that patients showing a RASZ shorter than 10 mm had a significantly higher risk of developing a type 1A endoleak [OR]: 9.63 [16]; however, this was a case–control study that involved patients treated with multiple stent graft models and did not consider anatomic parameters not related to the PSZ or periprocedural data. Current guidelines recommend imaging restriction for 5 years following the first CTA, which should be performed one month after the procedure. In total, 85 patients from our cohort had a circumferential aortic coverage shorter than 10 mm, 23 of them (27%) developed a type 1A endoleak, and 44 patients had a circumferential aortic coverage shorter than 5 mm but 11 of them developed a type 1A endoleak (25%). Our data suggest that the first RASZ measurement may be not sufficient to adequately stratify patients more at risk for type 1A endoleaks with adequate reliability to justify CTA restriction for 5 years, and when evaluating the individual risk of each patient, the model of endograft used should be considered. The observed difference in aneurysm size between groups should be further investigated in future research to assess its potential impact on sealing zone effectiveness and EVAR outcomes. We also found that patients presenting with a type 2 or 1b endoleak later in the follow-up also had a shorter RASZ. The observed association between proximal sealing zone parameters and type IB and II endoleaks may reflect the indirect effects of sealing zone insufficiency on the endograft’s long-term stability. However, this hypothesis requires further investigation, as these findings were not the study’s primary focus and are exploratory. The higher percentage of graft misplacement in the endoleak group and the higher difference between TASZ and RASZ should not be treated as prognostic factors, as they are not directly correlated with the absolute RASZ values. This study did not focus on the TASZ versus RASZ difference as a causative factor for type 1A endoleaks. Instead, it emphasized the systematic reduction in RASZ compared to TASZ, advocating for its recognition as a key metric in clinical planning and follow-up for EVAR procedures. Further studies with more advanced imaging techniques are needed to clarify whether there is any unseen blood spilling in the aneurysmal sac in patients with a short RASZ. High-resolution cinematic CTA, contrast ultrasonography, and computed fluid dynamics simulation could help elucidate RASZ stability and dynamics during the cardiac cycle and evolution.

### Study Limitations

This study’s limitations include a retrospective sample and a potential selection bias caused by the lack of sufficient CTAs for many patients. Another study limitation was the exclusive use of CTA to evaluate our patients. While the observed 5.5 mm cutoff for RASZ length appeared significant, the low number of events limited the ability to draw definitive conclusions. As the analysis was not designed directly to address this scope, this finding should be considered hypothesis-generating and warrants further validation in larger studies.

## 5. Conclusions

This study focused on introducing a practical and reproducible method for evaluating the planned and achieved proximal sealing zones (TASZs and RASZs) after EVAR. This study highlighted a significant discrepancy between the anticipated (TASZ) and achieved (RASZ) sealing zones in EVAR, demonstrating that the RASZ was systematically smaller even when the stent graft was optimally positioned, and hinted at the importance of assessing the real achieved sealing zone to identify patients at increased risk of adverse outcomes. The Kaplan–Meier survival analysis found relevance of the 5.5 mm cutoff, providing a potential parameter for risk stratification. Identifying an RASZ cutoff of 5.5 mm for type 1A endoleaks is an exploratory finding and requires further validation in future research. While the methodology provides valuable insights, additional research is needed to explore its broader applicability across different stent graft models and refine its integration into routine EVAR follow-up protocols.

## Figures and Tables

**Table 1:** Baseline demographics and comorbidities. Continuous variables are presented as mean ± SD, and categorical variables are presented as counts and percentages.

	Overall (275)	Group 1 (No Endoleak)	Group 2 (Any Endoleak)	*p*-Value (<0.05)
Age (years, SD)	72 ± 8	72 ± 8	73 ± 9	0.258
Female sex	21 (8.1%)	17 (8.5%)	4 (6.5%)	0.590
Diabetes	43(18.4%)	33 (18.4%)	10 (18.1%)	0.966
Arterial hypertension	225 (82%)	191 (81.6%)	46 (83%)	0.659
Dyslipidemia	147 (62.8%)	115 (64.2%)	32 (58.2%)	0.416
Cardiac disease	105 (44.8%)	81 (45.2%)	24 (43.6%)	0.833
Previous myocardial infarction	69 (29.5%)	53 (29.6)	16 (29.1)	0.941
Chronic obstructive pulmonary disease	125 (53.4%)	96 (53.6%)	29 (52.7%)	0.906
Chronic kidney disease	48 (20.5%)	35 (19.5%)	13 (23.6%)	0.512
Emergency setting	10 (4.1%)	6 (3.2%)	4 (6.9%)	0.208

**Table 2:** Baseline anatomic features. Continuous variables are presented as mean ± SD or median (IQR). * Statistically significant.

	Overall	Group 1 (No Endoleak)	Group 2 (Any Endoleak)	*p*-Value (<0.05)
AAA diameter (mm)	58 (51–67)	57 (50–64)	67 (56–78)	<0.001 *
Aortic bifurcation diameter (mm)	28 (24–38)	27 (23–35)	33(27–48)	<0.001 *
Access common iliac artery diameter (mm)	15 (13–21)	14 (12–21)	17 (13–22)	0.081
Access external iliac artery diameter (mm)	9.5 ± 3.8	9 (8–10)	10 (8.7–11)	0.011 *
Access femoral artery diameter (mm)	10.8 ± 4.2	10 ± 2.2	10.5 ± 2.2	0.085
Lowest renal to aortic bifurcation length (mm)	114 ± 19.8	112 (102–124)	116 (100–130)	0.623
Number of patent lumbar arteries	3 (2–4)	3 (2–4)	3 (2–4)	0.256
Infrarenal neck angulation	28 ± 19	27 ± 20	33 ± 20	0.097
Neck diameter (mm)	24.6 ± 4	24 ± 3	25 ± 5	0.540

**Table 3:** Periprocedural data. Continuous variables are presented as mean ± SD or median (IQR), and categorical variables are presented as counts and percentages. * statistically significant.

	Overall	Group 1 (No Endoleak)	Group 2 (Any Endoleak)	*p*-Value (<0.05)
Length of stay (days)	4 (3–5)	4 (3–5)	5 (3.5–6)	0.882
Procedure duration (hours)	3 ± 1.6	3.2 ± 1.3	3.4 ± 2.3	0.228
Sac embolization	57 (22%)	39 (15%)	18 (6.9%)	0.121
Complications	19 (7.3%)	12 (4.6%)	7 (11.3%)	0.167
Number of Endurant components	3 (2–3)	2 (2–3)	3 (2–3)	0.003 *
Bell-bottom component	53 (19%)	34 (17%)	19 (9.3%)	0.047 *
Proximal oversize	1.14 (1.06–1.23)	1.14 (1.06–1.24)	1.12 (1.05–1.23)	0.381

**Table 4:** Outcomes and sac regression including all patients. Continuous variables are presented as median (IQR), and categorical variables are presented as counts and percentages.

	Overall (275)
Follow-up time (months)	64 (31–106)
Any endoleak	62 (22.6%)
-Type 1A	24 (8.7%)
-Type 1B	11 (4%)
-Type 2	27 (9.8%)
Graft thrombosis	7 (2.6%)
Procedure-related reintervention	34 (12.3%)
Aneurysm-related mortality	5 (1.82%)
All-cause mortality	71 (25.8%)
Sac regression at more than one year:	
≥10 mm	126 (45.7%)
≥5 mm	182 (66.4%)
<5 mm/stable	51 (18.7%)
Growth > 5 mm	36 (13.1%)

**Table 5:** Target anticipated sealing zone vs. real achieved sealing zone. * Statistically significant.

	Preoperative Assessment	Postoperative Assessment	*p*-Value (<0.05)
Sealing area (mm^2^)	106 (50.0–210.1)	78 (34–155)	<0.001 *
Infrarenal area (mm^2^)	37.8 (31.5–47.4)	38.1 (31.5–45.9)	0.530
Proximal sealing zone length corrected for graft misplacement (mm)	15 (7–30)	11 (4–22)	0.001 *
Most distal point of neck/stent apposition area (mm^2^)	43.1 (36.1–52.8)	40.6 (33.4–51.9)	0.374

**Table 6:** Expected and achieved PSZ component comparisons between the two groups. * Statistically significant.

	Overall	Group 1 (No Endoleak)	Group 2 (Endoleak)	*p*-Value (<0.05)
Target anticipated sealing zona area (mm^2^)	106 (505–210)	114.2 (50–248)	65.0 (50.0–109.9) 60.0 (52.3–68.1) if type 1a splits groups	0.0024 * 0.10 if type 1a splits groups
Real achieved sealing zone area (mm^2^)	78.3 (34–155)	81.4 (34–154)	64.1 (27.2–158) 40.3 (23.6–71.7) if type 1a splits groups	0.831 0.110 if type 1a splits groups
Infrarenal area (mm^2^)	36.4 (30.8–54.9)	36.4 (30.6–45.9)	36.2 (32.3–43.4)	0.651
Distance between infrarenal level and most distal point of guideline neck (mm)	15 (7–30)	15.5 (7–33) 15 (7–30) if type 1a splits groups	10 (7–13) 8 (8–11) if type 1a splits groups	0.012 * 0.029 * if type 1a splits groups
Most distal point of guideline neck area (mm^2^)	43.1 (36.1–52.8)	42.9 (35.8–51.9) 42.8 (35.7–52.5) if type 1a splits groups	42.7 (36.0–54.2) 45.1 (38.1–61.4) if type 1a splits groups	0.751 0.191 if type 1a splits groups
Distance between infrarenal level and most distal point of circumferential stent apposition (mm)	13.5 (5.2–24)	15 (7–33) 15 (7–30) if type 1a splits groups	10 (7.7–13) 8 (8–11) if type 1a splits groups	0.006 * 0.029 * if type 1a splits groups
Distance circumferentially covered by endograft (mm)	11 (4–22)	12 (4–22) 12 (4–22) if type 1a splits groups	6 (3–20) 2.5 (0.5–10) if type 1a splits groups	0.155 0.027 * if type 1a splits groups
Most distal point of circumferential stent apposition area (mm^2^)	40.6 (33.4–52.0)	39.9 (33.0–51.3) 40.9 (33.5–52.1) if type 1a splits groups	46.2 (34.6–62.4) 36.6 (25.7–47.8) if type 1a splits groups	0.855 0.191 if type 1a splits groups
Proximal ovesizing	1.15 ± 0.12	1.15 ± 0.122	1.16 ± 0.12	0.186
Low graft misplacement (mm)	0 (0–2)	0 (0–1) the same if type 1a splits groups	0 (0–2) 2 (0–5) if type 1a splits groups	0.245 0.026 * if type 1a splits groups

**Table 7:** Univariable COX proportional hazard regression models for the risk of type 1A endoleak. * Statistically significant.

	HR	95% CI	*p*-Value (<0.05)
TASZ area	0.998	0.998–0.999	0.001 *
RASZ area	0.999	0.999–1.000	0.028 *
TASZ length	0.888	0.827–0.952	<0.001 *
RASZ length	0.910	0.855–0.969	0.003 *
Lost RASZ length	1.050	0.985–1.119	0.131
AAA diameter	1.021	1.001–1.042	0.041 *
Aortic bifurcation diameter	1.015	0.986–1.044	0.317
Infrarenal neck angulation	1.022	1.001–1.043	0.043 *

**Table 8:** Multivariable COX proportional hazard regression models for the risk of type 1A endoleak. * Statistically significant.

	HR	95% CI	*p*-Value (<0.05)
TASZ model			
TASZ length	0.877	0.804–0.955	0.003 *
AAA diameter	1.001	0.972–1.032	0.924
Infrarenal neck angulation	1.028	0.972–1.032	0.062
RASZ model			
RASZ length	0.921	0.860–0.987	0.020 *
AAA diameter	1.019	0.991–1.048	0.186
Infrarenal neck angulation	1.024	1.000–1.049	0.054

## Data Availability

All data generated or analyzed during this study are included in this published article. Further inquiries can be directed to the corresponding authors.
